# Efficient DNA barcode regions for classifying *Piper* species (Piperaceae)

**DOI:** 10.3897/phytokeys.70.6766

**Published:** 2016-09-20

**Authors:** Arunrat Chaveerach, Tawatchai Tanee, Arisa Sanubol, Pansa Monkheang, Runglawan Sudmoon

**Affiliations:** 1 Department of Biology, Faculty of Science, Khon Kaen University, Khon Kaen 40002, Thailand; 2 Genetics and Environmental Toxicology (GET) Research Group, Khon Kaen University, Thailand; 3 Faculty of Environment and Resource Studies, Mahasarakham University, Mahasarakham 44150, Thailand; 4 Faculty of Law, Khon Kaen University, Thailand

**Keywords:** DNA barcoding, *matK* gene, Piper species, *psbA*-*trnH* spacer, *rbcL* gene

## Abstract

*Piper* species are used for spices, in traditional and processed forms of medicines, in cosmetic compounds, in cultural activities and insecticides. Here barcode analysis was performed for identification of plant parts, young plants and modified forms of plants. Thirty-six *Piper* species were collected and the three barcode regions, *matK*, *rbcL* and *psbA*-*trnH* spacer, were amplified, sequenced and aligned to determine their genetic distances. For intraspecific genetic distances, the most effective values for the species identification ranged from no difference to very low distance values. However, *Piper betle* had the highest values at 0.386 for the *matK* region. This finding may be due to *Piper betle* being an economic and cultivated species, and thus is supported with growth factors, which may have affected its genetic distance. The interspecific genetic distances that were most effective for identification of different species were from the *matK* region and ranged from a low of 0.002 in 27 paired species to a high of 0.486. Eight species pairs, *Piper kraense* and *Piper dominantinervium*, *Piper magnibaccum* and *Piper kraense*, *Piper phuwuaense* and *Piper dominantinervium*, *Piper phuwuaense* and *Piper kraense*, *Piper pilobracteatum* and *Piper dominantinervium*, *Piper pilobracteatum* and *Piper kraense*, *Piper pilobracteatum* and *Piper phuwuaense* and *Piper sylvestre* and *Piper polysyphonum*, that presented a genetic distance of 0.000 and were identified by independently using each of the other two regions. Concisely, these three barcode regions are powerful for further efficient identification of the 36 *Piper* species.

## Introduction

Plants in the genus *Piper* have been used since prehistoric times for a variety of human activities. They are used as spices, in traditional and processed forms of medicines, in cosmetic compounds, in cultural activities and as insecticides (Chaveerach et al. 2006a, Scott et al. 2008, Fan et al. 2011). *Piper betle*, the betel plant, is one of the most important and well-known species of the genus. It contains important chemical substances, such as chavicol, cineol and eugenol, used in essential oils, medicines and insecticides (Yusoff et al. 2005, Misra et al. 2009). Eugenol has been reported as having anti-oxidant and anti-inflammatory properties (Misra et al. 2009). Although the betel plant is of great economic importance, it is challenging to cultivate. The main problem is foot and leaf rot, which is caused by the fungus *Phytophthora parasitica* Dast. In addition, the plant is subject to leaf spot, which is caused by bacteria (Silayoi et al. 1985, Banka and Teo 2000). Investigations of the genus *Piper* in Thailand (Chaveerach et al. 2008, 2009) have found that among the 43 *Piper* species, some produce a betel-like scent. Of these, all are wild species and hardy, producing numerous branches and leaves. They are tolerant and resistant to disease. Some produce a stronger scent than betel. Therefore, these species might be equally or more economically beneficial than the betel plant. The assured advantage is that there would be more choices of plants for use (Sanubol et al. 2014). Medicinal plants have been used in natural and modified forms. The modified forms such as dried sliced plant parts, powder and capsules, are difficult to recognize by physical features. Therefore, reliable identification methods for these plant forms should be developed. DNA barcoding is the most reliable and applicable method for identification. The method was developed in 2003 (Hebert et al. 2003). It principally uses short DNA sequences from appropriate genome regions for the identification of organisms. The *CO1* and 16s rDNA regions have been successfully used for most animals. For example, Hebert et al. (2004) used the mitochondrially encoded cytochrome c oxidase I (MT-CO1) to discriminate between bird species. Zhang and Hanner (2012) used sequences of *MT-CO1*, 16s RNA, *MT-CYB* and RNA 18s in 242 species of fish and in 11 *Epinephelus* species.

For plants, however, it is more of a challenge. Currently, several research groups are seeking a suitable genome region, and this effort has led to the identification of appropriate regions for DNA barcoding in some plant groups, such as the *matK* gene (Siripiyasing et al. 2012, Tanee et al. 2012), the *rbcL* gene (Tanee et al. 2012, Kwanda et al. 2013), the *psbA*-*trnH* spacer region (Chaveerach et al. 2011).

The standard barcodes used for most investigations of plants are the three plastid barcodes, which include *matK* gene, *rbcL* gene and *psbA*-*trnH* spacer, and one nuclear (ITS) regions identified by the CBOL Plant Working Group (2009), Chaveerach et al. (2011), Hollingsworth et al. (2011) and Monkheang et al. (2011). With the importance of *Piper* species as economically valued plants worldwide and with the plant parts of many species being used, such as the trunk, leaves and fruits, as well as young plants and processed plant materials in the forms of powder and slices, identifying the species used is paramount to verify the authenticity of such goods. Therefore, these products should have a specific marker that identifies a species using barcode for each species.

The aim of this research was to construct barcodes for *Piper* species in Thailand using *matK*, *rbcL* and the *psbA*-*trnH* spacer regions, as these species are important medicinal plants that have not been fully explored for barcode identification. Here we initiate the development of reference barcodes for plant parts, young plants and plant products.

## Materials and methods

### Plant materials

Species and sites of *Piper* recently reported in Thailand (Chaveerach et al. 2006a, 2006b, 2007, 2008, Sudmoon et al. 2011) were collected and carefully identified followed the literatures. Leaf samples were kept on ice, transferred to the laboratory, and then stored at -20 °C until further use.

### DNA extraction

Whole genomic DNA was extracted using a Plant Genomic DNA Extraction Kit (RBC Bioscience) following the kit protocols.

### Amplification of barcode fragments


 Polymerase chain reaction (PCR) analyses were performed with primer pairs (5'–3') ATCCATCTGGAAATCTTAGTTC and GTTCTAGCACAAGAAAGTCG (CBOL Plant Working Group 2009) for the *matK* gene, GTCACCACAAACAGAGACTAAAGC and GTAAAATCAAGTCCACCRCG (CBOL Plant Working Group 2009) for the *rbcL* gene, and GTTATGCATGAACGTAATGCTC and CGCGCATGGTGGATTCACAATCC (Hollingsworth et al. 2011) for the *psbA*-*trnH* spacer region. The reaction mixture (30 µl) consisted of 1× GoTaq Green Master Mix (Promega), 0.5 µM primers, and 30 ng of DNA template. The amplification profile included pre-denaturation at 94 °C for 1 min, 35 cycles of denaturation at 94 °C for 30 s, annealing at 52 °C (for *matK*) or 55 °C (for *rbcL* and the *psbA*-*trnH* spacer) for 30 s, extension at 72 °C for 1 min and a final extension at 72 °C for 5 min. The amplified products were subjected to 2% agarose gel electrophoresis.

### DNA sequencing and sequences analyses

The specific fragments amplified were sequenced at the DNA Sequencing Unit, Faculty of Medicine, Ramathibodi Hospital, Bangkok, Thailand. The sequences were then analyzed using Blast tools (http://blast.ncbi.nlm.nih.gov/Blast.cgi). Sequences were aligned for each genome region amplified to determine genetic distance values by MEGA6 (Tamura et al. 2013) using the Maximum Composite Likelihood method and are in the units of the number of base substitutions per site. Codon positions included were 1^st^+2^nd^+3^rd^+Noncoding. All positions containing gaps and missing data were eliminated. The sequences were submitted to GenBank and corresponding accession numbers were given.

## Results

Thirty-six *Piper* species were collected to construct barcodes. Because most of the *Piper* species that we investigated were wild, it was difficult to collect a sufficient amount of samples from all 36 *Piper* species to adequately construct barcodes. Sufficient samples were obtained for four species, *Piper nigrum*, *Piper betle*, *Piper sarmentosum* and *Piper retrofractum*, which are all economic plants.

The amplification of barcode bands from the *matK* region was not successful in two species, including *Piper montium* and *Piper rubroglandulosum* (♀). This may be because the DNAs were fragmented at the primer regions. Table 1 shows the GenBank accession numbers corresponding to 119 sequences from the *matK*, *rbcL* and *psbA*-*trnH* spacer regions for all 36 species studied.

**Table 1.** GenBank accession numbers of DNA barcoding from three regions of *Piper* species.

Scientific name	GenBank accession number^#^		
	*matK*	*psbA*-*trnH* spacer	*rbcL*
*Piper argyritis*	KM073990	JX442927, KM055176	JX291978, KM055126
*Piper betle* (♀)	GU372747, KM098143	GQ891996, JQ248053	JQ248074
*Piper betle* (♂)	KM098144	JQ248050	JQ248071
*Piper betloides*	KM098135	JQ248051	JQ248072
*Piper boehmeriifolium*	KM073991, KM073992	KM055177, KM055178	KM055127, KM055128
*Piper caninum*	KM073993, KM073994	KM055179, KM055180	KM055129, KM055130
*Piper colubrinum*	GU372751, KM073995	GQ892000	KM055131
*Piper crocatum*	KM098136	JQ248047	JQ248068
*Piper dominantinervium*	KM073996, KM073997	KM055181, KM055182	KM055132, KM055133
*Piper hongkongense*	KM073998, KM073999	KM055183, KM055184	KM055134, KM055135
*Piper khasianum*	KM074000, KM074001	KM055185, KM055186	KM055136, KM055137
*Piper kraense*	KM074002, KM074003	KM055187, KM055188	KM055138, KM055139
*Piper longum*	KM074004, KM074005	KM055189, KM055190	KM055140, KM055141
*Piper maculaphyllum*	KM074006, KM098137	JQ248046, KM055191	JQ248067, KM055142
*Piper magnibaccum*	KM074007, KM074008	KM055192, KM055193	KM055143, KM055144
*Piper montium*	n/a	KM055194, KM055195	KM055145, KM055146
*Piper mutabile*	KM074035	KM055196, KM055197	KM055147, KM055148
*Piper nigrum*	KM074009, KM074010	GQ891994, KM055198, KM055199	KM055149, KM055150
Piper pedicellatum var. eglandulatum	KM074011, KM074012	KM055200, KM055201	KM055151, KM055152
*Piper pendulispicum* (♀)	KM074013, GU372748	KM055202, GQ891997	KM055153, JX291979
*Piper phuwuaense*	KM074014, KM074015	KM055203, KM055204	KM055154, KM055155
*Piper pilobracteatum*	KM074016, KM074017, KM074018, KM074019	KM055205, KM055206, KM055207, KM055208	KM055156, KM055157, KM055158, KM055159
*Piper polysyphonum*	KM074020, KM074021	KM055209, KM055210	KM055160, KM055161
*Piper protrusum*	KM074032, KM074033	GU980900, KM055223	KM055172, KM055173
*Piper retrofractum*	GU372749, KM074034	GQ891998, KM055224	KM055175
*Piper ribesioides*	GU372750, KM074022	GQ891999, KM055211	KM055162
*Piper rubroglandulosum* (♀)	n/a	JX442926	JX291977
*Piper rubroglandulosum* (♂)	KM098138	JX442925	JX291976
*Piper sarmentosum*	GU372746, KM074023, KM074024	KM055212, KM055213	KM055163, KM055164
*Piper semiimmersum*	KM098139	JQ248045	JQ248066
*Piper submultinerve*	KM098140	JQ248048	JQ248069
*Piper sylvaticum*	KM074025, KM074026	KM055214, KM055215	KM055174
*Piper sylvestre*	KM074027, KM074028	KM055216, KM055217	KM055165, KM055166
Piper thomsonii var. trichostigma	KM074029	KM055218	KM055167
*Piper tricolor*	KM098141	JQ248049	JQ248070
*Piper umbellatum*	n/a	KM055219, KM055220	KM055168, KM055169
*Piper wallichii*	KM074030, KM074031	KM055221, KM055222	KM055170, KM055171
*Piper yinkiangense*	KM098142	JQ248052	JQ248073

# the sequence data deposited at www.ncbi.nlm.nih.gov/Genbank; n/a is "not amplified"

The intraspecific genetic distances for each region were the following: 1) for the *matK* region, the lowest value of 0.000 was observed in *Piper dominantinervium*, *Piper hongkongense*, *Piper kraense* and *Piper longum*, while the highest value of 0.386 was observed for *Piper betle*; 2) for the *rbcL* region, the lowest value of 0.000 was observed in *Piper dominantinervium*, *Piper hongkongense*, *Piper longum*, *Piper pedicellatum*, *Piper pilobracteatum*, *Piper polysyphonum*, *Piper sarmentosum*, *Piper sylvestre* and *Piper wallichii*, while the highest value of 0.166 was observed in *Piper betle*; 3) for the *psbA*-*trnH* spacer region, the lowest value of 0.000 was observed in *Piper dominantinervium*, *Piper khasianum*, *Piper kraense*, *Piper longum*, *Piper montium*, *Piper mutabile*, *Piper nigrum*, *Piper pilobracteatum*, *Piper polysyphonum* and *Piper sarmentosum* while the highest value of 0.117 was observed in *Piper boehmeriifolium*.

The interspecific genetic distances for each region were the following: 1) for the *matK* region the lowest value of 0.000 was observed in the paired species *Piper kraense* and *Piper dominantinervium*, *Piper magnibaccum* and *Piper kraense*, *Piper phuwuaense* and *Piper dominantinervium*, *Piper phuwuaense* and *Piper kraense*, *Piper pilobracteatum* and *Piper dominantinervium*, *Piper pilobracteatum* and *Piper kraense*, *Piper pilobracteatum* and *Piper phuwuaense* and *Piper sylvestre* and *Piper polysyphonum*, while the highest value of 0.486 was observed between *Piper ribesioides* and *Piper pilobracteatum*; 2) for the *rbcL* region, the lowest value of 0.000 was observed between pairs *Piper dominantinervium* and *Piper caninum*, *Piper kraense* and *Piper boehmeriifolium*, *Piper maculaphyllum* and *Piper khasianum*, *Piper magnibaccum* and *Piper khasianum*, *Piper magnibaccum* and *Piper caninum*, *Piper magnibaccum* and *Piper dominantinervium*, *Piper montium* and *Piper khasianum*, *Piper montium* and *Piper magnibaccum*, *Piper mutabile* and *Piper caninum*, *Piper mutabile* and *Piper dominantinervium*, *Piper mutabile* and *Piper magnibaccum*, *Piper nigrum* and *Piper caninum*, *Piper nigrum* and *Piper dominantinervium*, *Piper nigrum* and *Piper magnibaccum*, *Piper nigrum* and *Piper mutabile*, *Piper pedicellatum* and *Piper khasianum*, *Piper pedicellatum* and *Piper magnibaccum*, *Piper pedicellatum* and *Piper montium*, *Piper pedicellatum* and *Piper pendulispicum*, *Piper pendulispicum* and *Piper caninum*, *Piper pendulispicum* and *Piper dominantinervium*, *Piper pendulispicum* and *Piper magnibaccum*, *Piper pendulispicum* and *Piper mutabile*, *Piper pendulispicum* and *Piper nigrum*, *Piper phuwuaense* and *Piper caninum*, *Piper phuwuaense* and *Piper dominantinervium*, *Piper phuwuaense* and *Piper magnibaccum*, *Piper phuwuaense* and *Piper mutabile*, *Piper phuwuaense* and *Piper nigrum*, *Piper phuwuaense* and *Piper pedicellatum*, *Piper pilobracteatum* and *Piper caninum*, *Piper pilobracteatum* and *Piper mutabile*, *Piper polysyphonum* and *Piper khasianum*, *Piper polysyphonum* and *Piper magnibaccum*, *Piper polysyphonum* and *Piper montium*, *Piper sarmentosum* and *Piper longum*, *Piper sylvestre* and *Piper khasianum*, *Piper sylvestre* and *Piper magnibaccum*, *Piper sylvestre* and *Piper montium*, *Piper thomsonii* and *Piper nigrum*, *Piper pilobracteatum* and *Piper phuwuaense*, *Piper polysyphonum* and *Piper pendulispicum*, *Piper polysyphonum* and *Piper pedicellatum*, *Piper sylvestre* and *Piper pendulispicum*, *Piper sylvestre* and *Piper pedicellatum*, *Piper sylvestre* and *Piper polysyphonum*, *Piper wallichii* and *Piper umbellatum*, *Piper protrusum* and *Piper phuwuaense*, and *Piper protrusum* and *Piper pilobracteatum*, while the highest value of 0.213 was observed in the *Piper betle* and *Piper argyritis* pair; 3) for the *psbA*-*trnH* spacer region the lowest value of 0.000 was observed in the pairs of *Piper montium* and *Piper magnibaccum*, *Piper pilobracteatum* and *Piper caninum*, *Piper polysyphonum* and *Piper pedicellatum*, *Piper ribesioides* and *Piper pedicellatum*, *Piper sarmentosum* and *Piper longum*, *Piper sylvestre* and *Piper pedicellatum*, *Piper wallichii* and *Piper khasianum*, *Piper wallichii* and *Piper pedicellatum*, *Piper protrusum* and *Piper magnibaccum*, *Piper sylvestre* and *Piper polysyphonum*, *Piper sylvestre* and *Piper ribesioides*, *Piper wallichii* and *Piper polysyphonum*, *Piper wallichii* and *ribesioides*, *Piper wallichii* and *Piper sylvestre*, and *Piper yinkiangense* and *Piper betle*, while the highest value of 0.228 was observed between *Piper semiimmersum* and *Piper umbellatum*.

The genetic distance of the *matK* region in Table 2 is a representative example.

**Table 2.** Genetic distance values of the *matK* region for *Piper* identification, a representative example.

	*Piper argyritis*	*Piper betle*	*Piper betle*	*Piper betle*	*Piper betloides*	*Piper boehmeriifolium*	*Piper boehmeriifolium*	*Piper caninum*	*Piper caninum*	*Piper colubrinum*	*Piper colubrinum*	*Piper crocatum*	*Piper dominantinervium*	*Piper dominantinervium*	*Piper hongkongense*	*Piper hongkongense*	*Piper khasianum*	*Piper khasianum*	*Piper kraense*	*Piper kraense*	*Piper longum*	*Piper longum*	*Piper maculaphyllum*	*Piper maculaphyllum*	*Piper magnibaccum*	*Piper magnibaccum*	*Piper nigrum*	*Piper nigrum*	*Piper pedicellatum*	*Piper pedicellatum*	*Piper pendulispicum*
*Piper argyritis*	.000																														
*Piper betle*	.323	.000																													
*Piper betle*	.076	.362	.000																												
*Piper betle*	.126	.386	.106	.000																											
*Piper betloides*	.249	.423	.262	.260	.000																										
*Piper boehmeriifolium*	.004	.323	.074	.124	.252	.000																									
*Piper boehmeriifolium*	.011	.325	.074	.126	.258	.009	.000																								
*Piper caninum*	.013	.328	.080	.132	.249	.009	.017	.000																							
*Piper caninum*	.013	.328	.080	.132	.249	.009	.017	.000	.000																						
*Piper colubrinum*	.020	.332	.087	.134	.267	.017	.022	.026	.026	.000																					
*Piper colubrinum*	.089	.375	.143	.195	.310	.085	.093	.093	.093	.072	.000																				
*Piper crocatum*	.054	.358	.106	.145	.267	.052	.056	.061	.061	.065	.130	.000																			
*Piper dominantinervium*	.004	.323	.072	.121	.252	.002	.007	.011	.011	.015	.087	.050	.000																		
*Piper dominantinervium*	.004	.323	.072	.121	.252	.002	.007	.011	.011	.015	.087	.050	.000	.000																	
*Piper hongkongense*	.013	.321	.080	.130	.260	.011	.011	.020	.020	.024	.095	.059	.009	.009	.000																
*Piper hongkongense*	.013	.321	.080	.130	.260	.011	.011	.020	.020	.024	.095	.059	.009	.009	.000	.000															
*Piper khasianum*	.007	.325	.076	.126	.249	.002	.011	.011	.011	.020	.087	.054	.004	.004	.013	.013	.000														
*Piper khasianum*	.007	.325	.074	.124	.249	.004	.009	.013	.013	.017	.089	.052	.002	.002	.011	.011	.002	.000													
*Piper kraense*	.004	.323	.072	.121	.252	.002	.007	.011	.011	.015	.087	.050	.000	.000	.009	.009	.004	.002	.000												
*Piper kraense*	.004	.323	.072	.121	.252	.002	.007	.011	.011	.015	.087	.050	.000	.000	.009	.009	.004	.002	.000	.000											
*Piper longum*	.013	.325	.080	.130	.258	.011	.015	.020	.020	.024	.091	.054	.009	.009	.017	.017	.013	.011	.009	.009	.000										
*Piper longum*	.013	.325	.080	.130	.258	.011	.015	.020	.020	.024	.091	.054	.009	.009	.017	.017	.013	.011	.009	.009	.000	.000									
*Piper maculaphyllum*	.065	.369	.130	.171	.282	.065	.072	.074	.074	.080	.141	.111	.065	.065	.074	.074	.063	.063	.065	.065	.072	.072	.000								
*Piper maculaphyllum*	.054	.341	.072	.113	.249	.052	.054	.061	.061	.065	.130	.085	.050	.050	.059	.059	.054	.052	.050	.050	.054	.054	.111	.000							
*Piper magnibaccum*	.007	.325	.074	.124	.249	.004	.009	.013	.013	.017	.089	.052	.002	.002	.011	.011	.002	.000	.002	.002	.011	.011	.063	.052	.000						
*Piper magnibaccum*	.009	.323	.076	.124	.256	.007	.011	.015	.015	.015	.087	.054	.004	.004	.013	.013	.009	.007	.004	.004	.013	.013	.069	.054	.007	.000					
*Piper nigrum*	.015	.334	.087	.130	.262	.017	.022	.026	.026	.030	.102	.061	.015	.015	.024	.024	.020	.017	.015	.015	.022	.022	.072	.063	.017	.020	.000				
*Piper nigrum*	.009	.325	.080	.121	.249	.011	.015	.020	.020	.024	.095	.059	.009	.009	.017	.017	.013	.011	.009	.009	.017	.017	.072	.054	.011	.013	.020	.000			
*Piper pedicellatum*	.011	.328	.080	.126	.252	.007	.015	.015	.015	.024	.091	.054	.009	.009	.017	.017	.009	.011	.009	.009	.013	.013	.067	.046	.011	.013	.022	.013	.000		
*Piper pedicellatum*	.011	.325	.074	.124	.258	.009	.004	.017	.017	.022	.093	.056	.007	.007	.011	.011	.011	.009	.007	.007	.015	.015	.072	.054	.009	.007	.022	.015	.015	.000	
*Piper pendulispicum*	.004	.325	.076	.126	.252	.007	.011	.015	.015	.020	.091	.050	.004	.004	.013	.013	.009	.007	.004	.004	.009	.009	.067	.050	.007	.009	.013	.009	.009	.011	.000

## Discussion

Most of the 43 species of wild *Piper* in Thailand have many functional uses. Only four species, *Piper betle*, *Piper retrofractum*, *Piper nigrum* and *Piper sarmentosum* are economic and cultivated species, and all of these species are also used as ingredients in the products mentioned above in the introduction. *Piper betle* is a well-known species that is important for its chemical substances, including essential oils, chavicol, cineol and eugenol, which can be used for medicinal and insecticidal purposes. Because these plants are widely used, and used in several forms, which include plant parts, powdered preparations, capsule formulations and other preparations, their authenticity should be verified using DNA barcodes to establish the worthiness of these products for medicinal, cosmetics and house-hold use. To overcome the problems associated with identifying species based on morphological characters, DNA barcoding has been employed. For flowering plants in Thailand, the *psbA*-*trnH* spacer region was suggested as an efficient DNA barcode marker in *Senna* species (Monkheang et al. 2011), as well as *Smilax* and *Cissus* species (Kritpetcharat et al. 2011). In addition, the *rbcL* gene has been suggested as a marker in parasitic plants, including *Scurrula*, *Dendrophthoe*, *Helixanthera*, *Macrosolen* and *Viscum* species (Kwanda et al. 2013) and the *matK* gene marker was identified in some medicinal *Piper* species (Sudmoon et al. 2012). Therefore the authors selected these three regions for barcode promising in the *Piper* species.

The results from DNA barcoding 36 *Piper* species using three different marker regions support a previous hypothesis of genetic distance values (Hebert et al. 2003), showing a significant variance in sequences between species and a comparatively small variance within species. Note that the economic and planted species, *Piper betle* had the highest intraspecific genetic distance values of 0.386 for the *matK* region, which may have been due to the presence of human growth factors. The interspecific genetic distances for the *matK* region were effective for the identification of different species with 27 pairs of species ranging from a low of 0.002 to a high of 0.486, as shown in Table 2 and eight unidentified species pairs had a genetic distance of 0.000. This result agrees with the study by Hao et al. (2013) who claimed that *matK* had high species identification reliability and suggested that this region should be used for identification of *Piper* species along with the ITS region. Additionally, the *rbcL* and *psbA*-*trnH* spacer regions are effective for further identification of the other eight species pairs as shown in Table 3. The lowest genetic distance value is 0.010 of the pair *Piper sylvestre* and *Piper polysyphonum* to the highest value 0.129 for the pair *Piper phuwuaense* and *Piper kraense* in *psbA*-*trnH* spacer region. It can be concluded that these three barcode regions are powerful for further efficient identification of the 36 *Piper* species.

**Table 3.** Interspecific genetic distance values for identification of the eight pairs *Piper* species by *rbcL* and *psbA*-*trnH* spacer sequences.

Pairs of species	*matK* region	*rbcL* region	*psbA*-*trnH* spacer region
*Piper kraense* and *Piper dominantinervium*	0.000	0.005-0.008	0.111-0.117
*Piper magnibaccum* and *Piper kraense*	0.000	0.008	0.1110-0.123
*Piper phuwuaense* and *Piper dominantinervium*	0.000	0.000-0.003	0.021-0.026
*Piper phuwuaense* and *Piper kraense*	0.000	0.005-0.008	0.021-0.129
*Piper pilobracteatum* and *Piper dominantinervium*	0.000	0.003	0.021
*Piper pilobracteatum* and *Piper kraense*	0.000	0.003	0.010-0.123
*Piper pilobracteatum* and *Piper phuwuaense*	0.000	0.003	0.016-0.021
*Piper sylvestre* and *Piper polysyphonum*	0.000	0.000	0.000-0.010

The results presented here support those of Newmaster et al. (2007), who proposed to use *matK* and the *psbA*-*trnH* spacer to identify Myristicaceae plants, Sudmoon et al. (2012) who recommended independent analysis of each barcode region, and CBOL Plant Working Group (2009) who proposed *rbcL* and *matK* as the core DNA barcode regions for land plants.

## References

[B1] Banka RA Teo SP (2000) *Piper betle*. In: van der Vossen HAM Wessel M (Eds) PROSEA 16 Stimulant Plants. Backhuys, Leiden, 135–140. doi: 10.1073/pnas.0905845106

[B2] CBOL Plant Working Group (2009) A DNA barcode for land plants. Proceedings of the National Academy of Sciences of the United State of America 106: 12794–12797. doi: 10.17348/era.4.0.223-231 10.1073/pnas.0905845106 PMC2722355 19666622

[B3] Chaveerach A Mokkamul P Sudmoon R Tanee T (2006a) Ethnobotany of the genus *Piper* (Piperaceae) in Thailand. Ethnobotany Research and Applications 4: 223–231.

[B4] Chaveerach A Sudmoon R Tanee T Mokkamul P (2006b) Three new species of Piperaceae from Thailand. Acta Phytotaxonomica Sinica 44: 447–453. doi: 10.1360/aps040163

[B5] Chaveerach A Sudmoon R Tanee T Mokkamul P (2008) The species diversity of the genus *Piper* from Thailand. Acta Phytotaxonomica et Geobotanica 59: 105–163.

[B6] Chaveerach A Sudmoon R Tanee T Mokkamul P (2009) The genus *Piper* in Thailand, 2nd edition Khon Kaen Kanpim Printing, Khon Kaen, 172 pp.

[B7] Chaveerach A Tanee T Sudmoon R (2011) Molecular identification and barcodes for the genus *Nymphaea*. Acta Biologica Hungarica 62: 328–340. doi: 10.1556/ABiol.62.2011.3.11 21840834 10.1556/ABiol.62.2011.3.11

[B8] Chaveerach A Tanomtong A Sudmoon R Tanee T Mokkamul P (2007) A new species and two new varieties of *Piper* (Piperaceae) from Thailand. Acta Phytotaxonomica et Geobotanica 58: 33–38.

[B9] Fan LS Muhamad R Omar D Rahmani M (2011) Insecticidal properties of *Piper nigrum* fruit extracts and essential oils against *Spodoptera litura*. International Journal of Agriculture and Biology 13: 517–522.

[B10] Hao C Wu H Fan R Yang J Wu G Ma T Qin X (2013) DNA barcoding in genus *Piper*. Chinese Journal of Tropical Crops 2013-05. http://en.cnki.com.cn/Article_en/CJFDTotal-RDZX201305012.htm

[B11] Hebert PDN Stoeckle MY Zemlak TS Francis CM (2004) Identification of birds through DNA barcodes. PLoS Biology 2: 1657–1663. doi: 10.1371/journal.pbio.0020312 10.1371/journal.pbio.0020312 PMC518999 15455034

[B12] Hebert PDN Cywinska A Ball SL De Waard JR (2003) Biological identifications through DNA barcodes. Proceedings of the Royal Society of London Series B 270: 313–321. doi: 10.1098/rspb.2002.2218 12614582 10.1098/rspb.2002.2218 PMC1691236

[B13] Hollingsworth PM Graham SW Little DP (2011) Choosing and using a plant DNA barcode. PLoS ONE 6: . doi: 10.1371/journal.pone.0019254 10.1371/journal.pone.0019254 PMC3102656 21637336

[B14] Kritpetcharat O Kritpetcharat P Daduang J Daduang S Suwanrungruang K Khemtonglang N Bletter N Sudmoon R Chaveerach A (2011) Using DNA markers and barcoding to solve the common problem of identifying dried medicinal plants with the examples of *Smilax* and *Cissus* in Thailand. Journal of Medicinal Plants Research 5: 3480–3487.

[B15] Kwanda N Noikotr K Sudmoon R Tanee T Chaveerach A (2013) Medicinal parasitic plants on diverse hosts with their usages and barcodes. Journal of Natural Medicines 67: 438–445. doi: 10.1007/s11418-012-0695-2 22864809 10.1007/s11418-012-0695-2

[B16] Misra P Kumar A Khare P Gupta S Kumar N Dube A (2009) Pro-apoptotic effect of the landrace Bangla Mahoba of *Piper betle* on *Leishmania donovani* may be due to the high content of eugenol. Journal of Medical Microbiology 58: 1058–1066. doi: 10.1099/jmm.0.009290-0 19528177 10.1099/jmm.0.009290-0

[B17] Monkheang P Sudmoon R Tanee T Noikotr K Bletter N Chaveerach A (2011) Species diversity, usages, molecular markers and barcode of medicinal *Senna* species (Fabaceae, Caesalpinioideae) in Thailand. Journal of Medicinal Plants Research 5: 6173–6181. doi: 10.5897/jmpr11.1075

[B18] Newmaster SG Fazekas AJ Steeves RAD Janovec J (2007) Testing candidates plant barcode regions in the Myristicaceae. Molecular Ecology Resources 8: 480–490. doi: 10.1111/j.1471-8286.2007.02002.x 10.1111/j.1471-8286.2007.02002.x 21585825

[B19] Sanubol A Chaveerach A Sudmoon R Tanee T Noikotr K Chuachan C (2014) Betel-like-scented *Piper* plants as diverse sources of industrial and medicinal aromatic chemicals. Chiang Mai Journal of Science 41: 1171–1181.

[B20] Scott IM Jensen HR Philogène BJR Arnason JT (2008) A review of *Piper* spp. (Piperaceae) phytochemistry, insecticidal activity and mode of action. Phytochemistry Reviews 7: 65–75. doi: 10.1007/s11101-006-9058-5

[B21] Silayoi I Krengpiem P Disthabanchong A (1985) Effectiveness of systemic fungicides for control of *Phytophthora parasitica* NK1 in foot rot of *Piper betle*. Thai Agricultural Research Journal 3: 104–107.

[B22] Sudmoon R Tanee T Chaveerach A (2011) *Piper protrusum* (Piperaceae), a new species from southern Thailand based on morphological and molecular evidence. Journal of Systematics and Evolution 49: 468–475. doi: 10.1111/j.1759-6831.2011.00148.x

[B23] Sudmoon R Tanee T Wongpanich V Bletter N Chaveerach A (2012) Ethnobotany and species specific molecular markers of some medicinal *sakhan* (*Piper*, Piperaceae). Journal of Medicinal Plants Research 6: 1168–1175.

[B24] Tamura K Stecher G Peterson D Filipski A Kumar S (2013) MEGA6: molecular evolutionary genetics analysis version 6.0. Molecular Biology and Evolution 30: 2725–2729. doi: 10.1093/molbev/mst197 24132122 10.1093/molbev/mst197 PMC3840312

[B25] Yusoff Z Mahmud Z Saleh SH Mohd EY (2005) Study on the chemical constituents of *Piper betle* L. in relation to their possible insect attractant property. Malaysian Journal of Science 24: 143–147.

[B26] Zhang JB Hanner R (2012) Molecular approach to the identification of fish in the South China Sea. PLoS ONE 7: . doi: 10.1371/journal.pone.0030621 10.1371/journal.pone.0030621 PMC3281855 22363454

